# Racial Differences in Perceived Risk and Sunscreen Usage

**DOI:** 10.7759/cureus.33752

**Published:** 2023-01-13

**Authors:** Rebecca Fliorent, Alicia Podwojniak, Lianne Adolphe, Katharine Milani

**Affiliations:** 1 Department of Molecular Biology, Rowan University School of Osteopathic Medicine, Stratford, USA

**Keywords:** prevention in primary care, primary care education, skin cancers, public health education, ethnic groups, healthcare inequality, melanoma, sunscreen, racial bias

## Abstract

Background

Although White individuals have higher incidence of melanoma, clinical outcomes are worse among patients with skin of color. This disparity arises from delayed diagnoses and treatment that are largely due to clinical and sociodemographic factors. Investigating this discrepancy is crucial to decrease melanoma-related mortality rates in minority communities. A survey was used to investigate the presence of racial disparities in perceived sun exposure risks and behaviors.

Methods

A survey consisting of 16 questions was deployed via social media to assess skin health knowledge. Over 350 responses were recorded, and the extracted data were analyzed using statistical software.

Results

Of the respondents, White patients were significantly more likely to have higher perceived risk of developing skin cancer, highest levels of sunscreen usage, and higher reported frequency of skin checks performed by primary care providers (PCPs). There was no difference between racial groups in the amount of education provided by PCPs related to sun exposure risks.

Conclusion

The survey findings suggest inadequate dermatologic health literacy as a result of other factors such as public health and sunscreen product marketing rather than as a consequence of inadequate dermatologic education provided in healthcare settings. Factors such as racial stereotypes in communities, implicit biases in marketing companies, and public health campaigns should be considered. Further studies should be conducted to determine these biases and improve education in communities of color.

## Introduction

Melanoma is one of the most common skin cancers in the United States, resulting in more than 9000 deaths per year [1]. Patients diagnosed early have a greater survival rate than those diagnosed after the cancer has already metastasized to the lymphatic system [2]. This makes time for treatment crucial in minimizing mortality. Although White patients have a higher incidence of melanoma, the overall survival rate of melanoma in patients of color is significantly lower due to delayed diagnosis and treatment [3]. The disparity in time to diagnosis and treatment in patients of color is due to a myriad of clinical, psychological, and sociodemographic factors, such as atypical presentation, decreased health literacy, provider supply, distance to dermatologic care, and poverty rate [4]. The commonly held belief that darker-skinned individuals are protected from melanoma is one of the most common barriers in diagnosing melanoma at earlier stages. Studies have shown that despite Black patients having a higher mortality rate when diagnosed with melanoma, they were less likely to be concerned with the threat of the disease and less likely to recognize malignant lesions [5]. Studies have confirmed that even the darkest skin tones are not completely protected against ultraviolet (UV) radiation. Even at very low UV exposures, DNA damage can be measured in all skin types, and this DNA damage may result in the development of melanoma [6].

It has also been proposed that the unfavorable clinical outcomes seen in patients with skin of color are less due to the aggression of the disease but may instead be due to lower socioeconomic status (SES), which impacts access to healthcare and subsequently time to diagnosis. Melanoma treatments are estimated to be approximately $44.9 million among Medicare patients with existing diagnoses and $932.5 million among new diagnoses [7]. Future projected costs of treating new patients with melanoma are expected to triple to about $1.6 billion by 2030 according to recent CDC data. Costs vary based on the stage of disease, the level of progression, and the type of care received. One study identified that the cost of melanoma treatment per patient averaged around $1000 in 2006 and has increased to $1600 in 2011 [8]. Given these staggering numbers, there is further incentive for promoting efforts to enhance primary and secondary prevention efforts. A study involving White, Hispanic, Black, and Asian patients with cutaneous melanoma demonstrated that low socioeconomic status (SES) independently predicted poor outcome among patients [9]. Advanced stages of the disease often require more aggressive treatments such as chemotherapy or surgery, which are costly modalities that tend to be seen more in minority populations [10]. Further, dermatologic provider density is disproportionately lower in low-income communities [11]. This results in greater responsibility for the primary care provider (PCP) to diagnose a condition that has a less common presentation in patients with skin of color, and oftentimes, they fall short. Such systemic disparities play an additional role in the inequity of melanoma diagnoses.

Ethnic minority populations have been widely underrepresented in dermatologic pedagogy, research, and public health marketing campaigns. As such, there are few studies investigating the racial disparities in skin protection and the role clinical, psychological, and sociodemographic factors play in the diagnosis and treatment of melanoma. The aim of this brief report is to examine trends related to skin cancer and related behaviors as they relate to health literacy and other risk factors in White versus non-White patients. The results from the survey data collected indicate that there may be social and cultural factors contributing to this disparity that is observed in melanoma outcomes in White versus non-White individuals. The results presented here may help redefine how healthcare practitioners educate the public on melanoma and sun protection behaviors.

## Materials and methods

To explore the relationships between race and skin health knowledge, a 16-question survey was deployed via Qualtrics to examine the presence of health disparities in melanoma diagnosis and treatment. The first four questions gathered socioeconomic and demographic data. There were five Likert scale questions that focused on sunscreen usage and reasons that may impact its use and six yes/no questions that attempted to obtain information on their experience with their primary care providers as it relates to skin conditions including melanoma and sunburns. There was also one open-ended question for any additional comments the participants wanted to include. The questions included in this survey were developed based on an extensive literature review and modeled after previously published surveys.

The survey was distributed on various social media sites including Facebook, Twitter, GroupMe, and Reddit, and it was made available during the summer months to attempt to capture peak sun protection usage. The social media groups targeted populations in and around the Southern New Jersey/Philadelphia suburban area since the survey included a question on which primary care provider group the participant utilized. A majority of the respondents selected “other” for the primary care question and did not fill in their provider information; thus, we did not include that information in our analysis. The demographic proportions of the participants reflect the makeup of this geographic area, with approximately 10%-20% of the population being non-White. The subjects were limited to individuals 18 years of age or older who completed the survey. After posting, the survey link remained active online for eight weeks.

Participants were asked to self-report their racial group (Black, White, Asian/Pacific Islander, or Hispanic), as well as other demographic information including age, sex, education level, and average household income. Information regarding the various aspects of sun exposure risks, education, and behaviors was collected using a combination of multiple-choice and yes/no questions. Questions asking about the frequency and severity of sunburns, past melanoma diagnosis (either the patient themselves or family history), the patient’s understanding of skin care (“How often do you use sunscreen?” and “What is your main reasoning for using sunscreen?”), and the level of education received on skin care from primary care physicians (“How often do you receive skin checks at your primary care office?” and “How much education have you received on melanoma from your healthcare provider?”) were all used to evaluate knowledge and perceived risk. To determine statistical significance, the responses of the racial groups for “yes/no” questions were compared utilizing a chi-square analysis. Multiple Student’s t-tests were performed to compare the average response rates between the racial groups for scaled questions.

## Results

**Figure 1:**
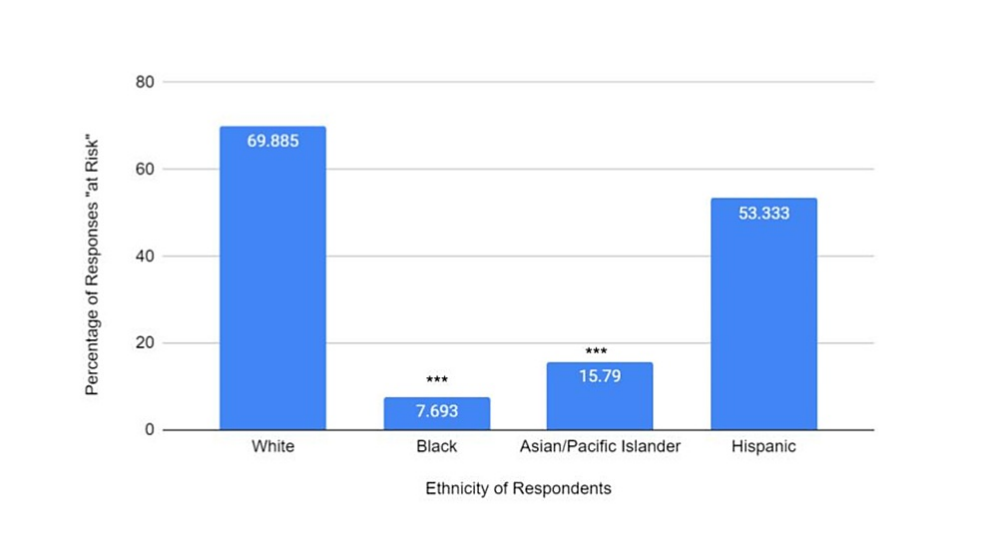
Perceived risk of UV-caused skin damage between racial groups Graph showing differences in perceived risk of developing UV damage, including skin cancer, between ethnic groups based on answers to “yes/no” questions. Total responses: White, 259; Black, 13; Asian/Pacific Islander, 19; and Hispanic, 15. There was a statistically significant difference found between Black and Asian/Pacific Islander groups when compared to White respondents, with those groups having a lower perceived risk. A chi-square analysis was performed to determine significance ***P value < 0.0001 UV: ultraviolet

A total of 306 individuals completed this survey, including 19 Asian/Pacific Islander participants, 13 Black participants, 15 Hispanic/Latino participants, and 259 White participants. These results reflect the demographic distributions seen in the Southern New Jersey/Philadelphia suburban area. The parameters analyzed were perceived risk of skin cancer, sunscreen usage, perceived education from primary care providers on skin health, perceived education of participants with and without a family history of melanoma, and skin checks performed. There was a significant difference in the perceived risk of skin cancer between ethnic groups when compared to White respondents (Figure 1).

**Figure 2:**
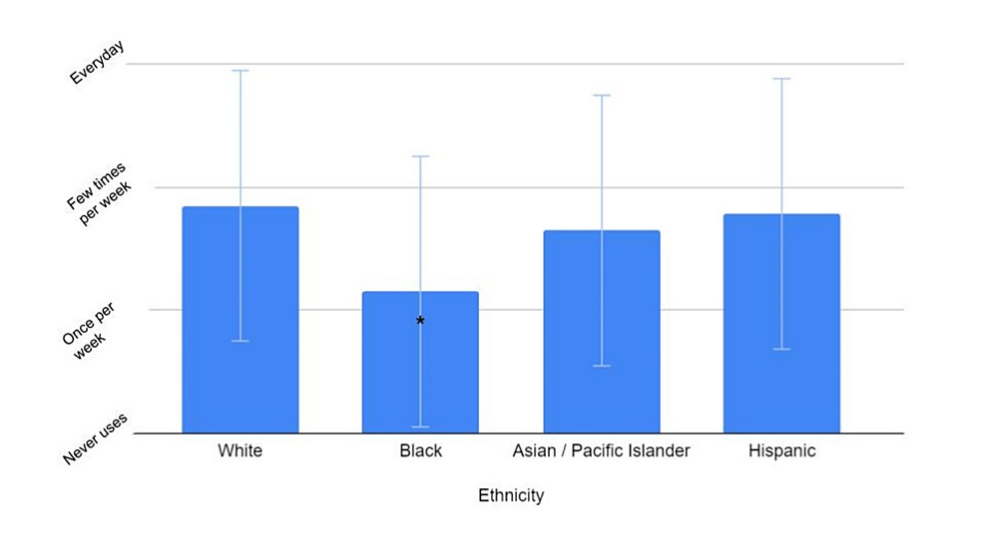
Average reported sunscreen use per week Graph showing the average reported sunscreen usage per week based on ethnicity. There is a statistically significant difference between the reported sunscreen usage of White and Black populations; error bars represent standard deviation. There is also a decrease between White and non-white groups, but it is not statistically significant (p value = 0.642) *P value < 0.05

In response to a “yes/no” question, Black and Asian/Pacific Islanders had the lowest perceived risk of being diagnosed with skin cancer than the other participants (P < 0.0001). Black participants also had significantly lower self-reported sunscreen usage when compared to White participants (P < 0.05) than those of the other groups (Figure 2).

**Figure 3:**
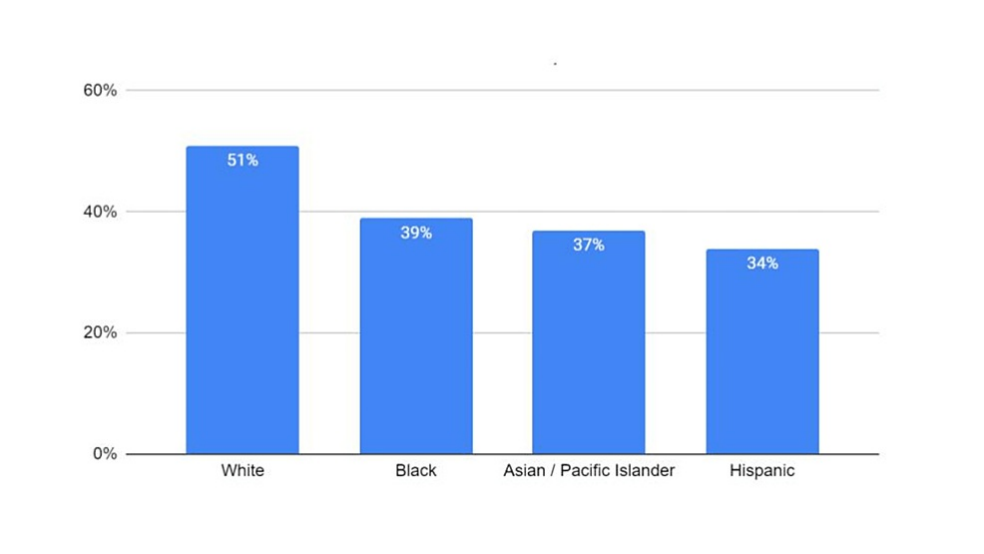
Percentage of respondents having received education on the risks of sun exposure Graph displaying the percentage of respondents who claim to have received education (either written or verbal) on the risks of sun exposure, including melanoma, from their primary care physician. Although there is an overall decrease, the difference between the White ethnic group and others was not significant (p value = 0.162)

Data on skin cancer education received from healthcare professionals showed that there was an overall decrease in the levels of reported education on the risks of sun exposure received between White and non-White ethnic groups (Figure 3).

A nonsignificant downward trend was observed, with Hispanic individuals reporting the lowest levels of received education.

## Discussion

Ethnic minorities with darker skin tones, inducing Black individuals, face a greater risk of melanoma-related mortality when compared to White populations [12]. The barriers hindering appropriate treatment and diagnosis of melanoma in these patients were explored in our survey and include the lack of health literacy and psychological and socioeconomic factors. Investigating these disparities is crucial in order to decrease the disparity in melanoma-related mortality rates.

Although there are significantly higher rates of melanoma among non-Hispanic White individuals, the cases among patients with darker skin are generally diagnosed at later stages and have worse clinical outcomes. This may be due to a variety of factors including differing clinical presentations in individuals with black and brown skin, various socioeconomic factors, and/or receiving different or no information from their primary care providers. The survey included questions designed to assess the impact of information about the risks of sun exposure received from primary care offices.

The results from the survey indicate that there is a slight decrease in the percentage of non-White individuals receiving this type of information from their primary care providers; however, this decrease does not reach statistical significance (Figure 3). Approximately 40% of Black individuals reported receiving information about the risks of sun exposure, while only 8% reported feeling at risk of skin damage as a result of sun exposure (Figure 3 and Figure 1). Black individuals also had the lowest reported sunscreen usage among the different ethnic groups (Figure 2). The difference between the perceived risk of sun exposure and the education received about those risks suggests that there may be more factors that heavily influence perceived risk and subsequent behaviors, such as sunscreen application. These findings point to a prodigious public health issue. Stereotypes surrounding skin health for patients with darker skin are perpetuated by a lack of dermatologic representation in the media, including sunscreen marketing, contributing to the Black population’s low health literacy on the efficacy of sunscreen use in preventing skin cancer.

The stereotypes surrounding darker skin and skin cancer impact risk perception. Theories in health psychology argue that people with a higher perceived risk of developing certain illnesses are more likely to engage in health protective behavior [13]. Black individuals are more likely to have inaccurate risk perceptions and less knowledge about skin cancer than White individuals, leading to poor health protective behaviors [14]. In one study assessing knowledge, attitudes, and practices regarding skin cancer and sun exposure, it was found that Black and African American men were less likely to know that people with dark skin could get skin cancer and did not feel that they were at risk of developing melanoma as compared to White participants [15]. In another study, this low perceived risk was confirmed, with 46% of African Americans reporting zero skin cancer risk and 76% perceiving zero or low risk [13].

Proper and regular application of sunscreen may help reduce the risk of developing melanoma; however, implicit bias and stereotyping present in the sun protection factor (SPF) marketing industry and cosmetic websites may have an effect on propagating erroneous ideologies concerning skin cancer in both ethnic minority populations and healthcare providers. These psychological phenomena inevitably act as a barrier, hindering diagnosis and treatment in patients of color. While skin health disparities may exist at a primary care level, we believe that this disparity stems from a larger public health issue. Educating at-risk minority groups on the potential dangers of skin cancer can lead to an increase in sun protective behaviors and potentially eliminate the disparities seen in diagnosis [16].

Historically, public health education and interventions to promote sun protective behaviors have been aimed toward the non-Hispanic White community despite Black individuals having a higher mortality rate. Studies have shown that sun protective behavior improved in Black participants who received melanoma education and that those improvements were long-lasting [17]. The SPF marketing industry and cosmetic websites display a lack of appropriate marketing geared toward protecting non-White individuals from skin cancer. One review identified the Internet and social media as top sources of skin health information in people of color [18]; however, another study identified websites that recommend more expensive and less protective sunscreens to consumers with skin of color, as compared to White consumers [19]. Social media marketing poses a clear route to obtaining skin health information and can influence the consumer’s understanding and decisions.

Limitations

Despite having canvassed a wide variety of participants, the results reported in this research have several limitations. A large percentage of the responses was obtained from White survey participants leading to a disproportionate response rate. This may have been due to the social media sites that were targeted for the survey distribution. One of the initial focuses of the survey was on healthcare systems in the Southern New Jersey/Philadelphia area, so the survey was distributed to social media groups that targeted those populations. The demographics of this geographic area are reflected in our survey results, with approximately 85% of the population being White. Nonetheless, the results in some categories obtained were statistically significant. Likewise, the self-reported data acquired from our survey do not allow for the verification of responses and can lead to a number of biases including selective memory and exaggeration. Lastly, the education level of the participants skewed the results of certain survey questions. A majority of patients reported that they obtained a college or doctorate degree leading to an uneven response to questions about previous knowledge on skin cancer and UV scales.

## Conclusions

Our findings confirm the existing disparities between different ethnic groups in their perception of risks associated with UV exposure and the development of melanoma. The results obtained in this study are in line with the perception of a reduced risk to traditionally darker skin tone ethnic groups likely resulting in a reduction in the amount of preventative action being taken in the form of sunscreen application that we also observed. Our results also indicate that this perception of a reduced risk of UV-caused skin damage is not due to the lack of information being received from primary care providers. It is encouraging to see that healthcare providers are providing appropriate education to their patients regardless of their ethnicity. However, this does indicate that there are other factors in play that are influencing this perceived risk and subsequent behaviors. Our hypothesis is that this perception is heavily influenced by marketing strategies, and those strategies are possibly negatively impacting the public health of minority ethnic groups. It is imperative to continue to educate the public about skin health in non-White individuals to further reduce their risk, and the media’s role in this public perception should be more closely investigated. It should be a public health initiative to change the narrative of skin health in ethnic minorities.
